# A Case of Esophageal Adenocarcinoma Metastatic to the Breast

**DOI:** 10.7759/cureus.54959

**Published:** 2024-02-26

**Authors:** Prashanth Kotla, Suimin Qiu, Jing He

**Affiliations:** 1 School of Medicine, University of Texas Medical Branch, Galveston, USA; 2 Pathology, University of Texas Medical Branch, Galveston, USA

**Keywords:** immunohistochemical stains, pathology, esophageal adenocarcinoma, breast metastases, extramammary malignancies

## Abstract

Metastatic carcinoma infiltrating the breast from extramammary malignancies is an infrequent occurrence. Extramammary carcinomas that may be metastatic to the breast include gastrointestinal cancers, ovarian cancer, and lung cancer, among others. These metastatic lesions often pose a diagnostic challenge, resembling primary breast cancer in both clinical and radiographic presentations. The incidence of esophageal cancer metastasizing to the breast is low. Esophageal cancer more commonly spreads to locoregional lymph nodes, lungs, liver, and bone. We present a unique case where primary esophageal adenocarcinoma metastasized to the breast. Our aim is to raise diagnostic awareness by delineating the clinical and pathological findings of this exceptionally infrequent disease.

## Introduction

While primary malignant breast tumors are the most common neoplasms in women, the occurrence of cancer metastasizing to breast cancers is exceedingly rare [1,2]. Non-breast primary tumors, originating from various sites such as the gynecological tract (uterus, cervix, ovary), genitourinary tract (bladder, kidney, prostate), gastrointestinal organs (colon, ileum, stomach), pancreaticobiliary organs, as well as the skin and parotid gland, have been reported [2].

Esophageal cancer stands as the sixth-leading cause of cancer-related fatalities worldwide [3]. Metastasis in esophageal cancer frequently involves non-regional lymph nodes, peritoneum, lungs, liver, bone, and brain [4,5]. The occurrence of esophageal cancer metastasizing to the breast is a rare phenomenon with an unclear etiology [6]. 

In this report, we present a case wherein primary esophageal adenocarcinoma, originating from background Barrett's esophagus, has metastasized to the breast. In such a case, differentiating metastatic carcinoma from primary breast cancer is crucial for formulating an appropriate treatment plan. A meticulous histological examination correlated with the patient’s medical history and complemented by immunohistochemical stains is essential for achieving an accurate diagnosis. 

## Case presentation

A 55-year-old female, with a medical history of gastroesophageal reflux disease and a smoking history of 30+ pack-years, presented to an outside healthcare facility due to a two-month history of dysphagia aggravated by the ingestion of solid foods. An upper GI endoscopy was performed, revealing distal esophageal stenosis, accompanied by a hiatal hernia and grade B esophagitis, per the Los Angeles classification system of gastroesophageal reflux disease. A subsequent biopsy of the stenotic area identified the anomaly as an invasive adenocarcinoma in the distal esophagus, secondary to Barrett esophagus. 

The patient came to our institution seeking a consultation with thoracic surgery, and a referral was made to the GI department for an endoscopic ultrasound to assist in staging the cancer. The endoscopic ultrasound revealed a hypoechoic mass located in the distal third of the esophagus, along with two anomalous lymph nodes in the distal paraoesophageal mediastinum. Subsequent staging via endoscopic ultrasound classified the esophageal adenocarcinoma as stage III, with a TNM classification of cT2, cN1, and cM0. The patient promptly initiated neoadjuvant chemoradiation (FOLFOX (folinic acid, fluorouracil, and oxaliplatin) regimen) with the intention of scheduling an esophagectomy in the future. 

**Figure 1:**
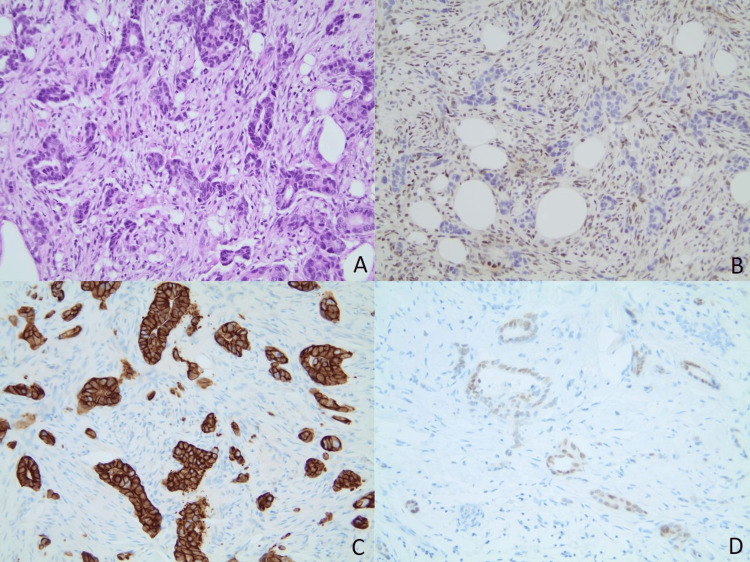
Histological findings of the core needle biopsy of the right breast. (A) H&E staining shows infiltrating malignant glands in the background of desmoplastic stromal reaction (magnification: 200x); (B) TRPS1 immunostaining shows negative nuclear stain in the tumor cells (magnification: 200x); (C) The immunostaining of CK7 demonstrates strong positivity in the tumor cells (magnification: 200x); (D) Tumor cells exhibit nuclear expression for CDX2 (magnification: 200x).

Post neoadjuvant therapy, a PET scan indicated stable disease. Subsequently, the patient underwent an esophagectomy, which revealed no residual carcinoma. Histological examination of 21 lymph nodes revealed no evidence of metastatic adenocarcinoma. Post-treatment tumor pathological staging was classified as ypT0, ypN0, ypMNA. During a surveillance CT scan of the chest/abdomen/pelvis six months later, an 11 mm nodular density was observed in the right breast. It is pertinent to note the patient's familial history of breast cancer involving both the mother and paternal half-sister, despite the patient's own Breast Cancer gene (*BRCA*) testing yielding negative results. An ultrasound-guided core needle biopsy of the right breast lesion (Figure 1) demonstrated an invasive adenocarcinoma that was negative for estrogen receptor (ER), progesterone receptor (PR), and human epidermal growth factor receptor 2 (Her2). This diagnosis occurred approximately one year after the patient's completion of esophageal cancer treatment, during a period when the patient was in remission. 

**Figure 2:**
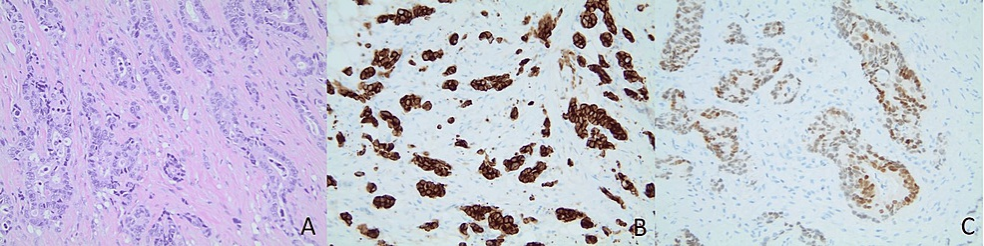
Histological findings of the excisional biopsy of the peritoneum. (A) H&E staining shows invasive adenocarcinoma (magnification: 200x); (B) CK7 immunostaining shows strongly diffuse positivity in the tumor cells (magnification: 200x); (C) The immunostaining of CDX2 shows positive nuclear staining (magnification: 200x).

Following the diagnosis of breast carcinoma, a subsequent abdomen/pelvis CT revealed multiple peritoneal nodules. Further investigation via diagnostic laparoscopy and excisional biopsy confirmed peritoneal carcinomatosis, likely indicating a concurrent relapse of the original esophageal adenocarcinoma. The abdominal lesions exhibited strong and diffuse positivity for CK7 and CDX2, confirming the diagnosis of metastatic esophageal adenocarcinoma (Figure 2). 

In comparison, the histological morphology of the tumor cells in the breast biopsy resembled that of the previous esophageal biopsy. The tumor cells of the breast biopsy were positive for CK7 and CDX2, while negative for TRPS1, mammaglobin, and GATA3 (Figure 1). Due to the absence of specific tumor markers typical of primary breast cancer, such as TRPS1, GATA3, and mammaglobin, it is unlikely that the breast lesion is a primary breast cancer. The findings strongly suggest metastatic esophageal adenocarcinoma involvement in the breast tissue. 

The treatment plan primarily targeted the relapsed esophageal adenocarcinoma while concurrently monitoring the breast carcinoma. The patient received eight cycles of chemotherapy involving a FOLFOX/Nivolumab regimen. A chest/abdomen/pelvis CT, conducted after the seventh cycle, indicated a stable disease state. However, the patient was admitted to the hospital shortly afterward due to aspiration pneumonia, which progressed to sepsis and respiratory arrest. After transitioning to end-of-life care, the patient, unfortunately, passed away due to complications related to pneumonia. 

## Discussion

A crucial aspect requiring thorough investigation involves discerning between a primary breast cancer and a secondary breast cancer metastasizing from the esophagus. This distinction holds particular significance for our patient, given her familial history of breast cancer in her mother and paternal half-sister. Despite the patient testing negative for *BRCA1* and *BRCA2*, it remains imperative to differentiate whether her breast carcinoma emerged independently within the breast tissue or if it metastasized from the primary esophageal lesion. This differentiation significantly impacts both the prognosis and the selection of appropriate treatment strategies. 

In our patient's case, the definitive evidence establishing breast cancer metastasis from the primary esophageal lesion was derived from the final surgical pathology report following the core needle biopsy. The metastatic esophageal adenocarcinoma identified in the peritoneum and breast exhibited positive testing for CK-7 and CDX2. The breast lesion tested negative for mammaglobin, GATA-3, and TRPS1. 

Mammaglobin, a protein typically present in mammary gland cells, shows elevated expression in primary breast cancers compared to noncancerous breast cells [7]. Studies by Watson et al. [7] and Wang et al. [8] demonstrated significant positive immunohistochemical staining for mammaglobin in 80% of breast ductal carcinomas and 76% of primary breast cancers with lymph node metastasis, respectively. 

GATA-3 functions as a transcription factor in mammary glands, aiding in their development and preserving the differentiation process of cells within the ducts' lumen [9]. It is frequently employed as an immunohistochemical marker to ascertain the primary origin of a lesion from breast tissue [9]. Cimino-Mathews et al. reported a 67% positivity rate for GATA-3 among tested invasive ductal carcinomas of primary breast origin, which declined to 43% among triple-negative invasive ductal carcinomas of primary breast origin [10]. 

TRPS1 (trichorhinophalangeal syndrome type 1) serves as a transcriptional repressor protein that demonstrates a robust indication of primary breast cancer origin [11]. While GATA3 exhibits high sensitivity in identifying tumors of primary breast origin, its sensitivity when it comes to pinpointing triple-negative breast cancers as originating from the breast is lower [12]. However, TRPS1 has shown remarkable sensitivity and specificity as a breast-specific tumor marker, particularly in identifying triple-negative (estrogen receptor (ER), progesterone receptor (PR), human epidermal growth factor receptor 2 (HER2)) breast cancers of primary origin [13]. Recent studies by Du et al. [12] and Ai et al. [13] noted an overall TRPS1 positivity rate of 92.3% and 91%, respectively, across various breast carcinoma types. Among various subtypes of triple-negative breast cancers, Du et al. [12], Ai et al. [13], and Yoon et al. [14] observed TRPS1 positivity rates of 91.2%, 86%, and 95%, respectively. Ai et al.'s study highlighted TRPS1's higher positivity rate compared to GATA3 in both metaplastic and non-metaplastic triple-negative breast cancers [13]. This same study reported minimal to absent TRPS1 expression in urothelial carcinomas, adenocarcinomas of the lung, colon, stomach, pancreas, and renal cell carcinoma, among others [13]. In our case, considering the breast carcinoma's triple-negative nature and its negative staining for TRPS1, it strongly supports its origin from the esophagus rather than the breast. 

CDX-2, a transcription factor, typically exhibits positive staining in gastrointestinal carcinomas, and it is not commonly associated with primary breast origin [15,16]. A comprehensive literature review in a recent study, encompassing 20 studies published between 2003 and 2017 examining CDX2 expression in primary and metastatic breast cancers, reported an overall CDX2 positivity rate of 0.7% [17]. In our case, both the breast carcinoma and the metastatic esophageal adenocarcinoma in the peritoneum displayed positive nuclear stains for CDX-2. The similarity between the identified peritoneal metastasis and the breast carcinoma strongly suggests that the breast lesion is secondary to esophageal metastasis rather than representing a primary breast neoplasm. The collective evidence from these immunohistochemical findings strongly supports the diagnosis of breast carcinoma secondary to the primary esophageal adenocarcinoma. 

In a case report by Miyoshi et al., the authors highlighted the rarity of metastatic breast tumors originating from extramammary sites, indicating that approximately 0.5-5.1% of all breast cancers stem from extramammary metastasis [18]. Autopsy series reported a higher incidence of extramammary metastasis detection, ranging from 1.7% to 6.6% [18]. Distinguishing between primary and metastatic breast cancer holds significant treatment implications since a breast metastasis would generally not require invasive surgical interventions, like radical breast mastectomy, typically undergone for primary tumors. Instead, the focus would lean more toward systemic treatments targeting the primary lesion [19]. 

Shiraishi et al. reported a case where poorly differentiated esophageal squamous cell carcinoma metastasized to the breast, initially misdiagnosed as primary breast cancer via core needle biopsy [19]. Subsequent to the initial diagnosis, the patient underwent a modified radical mastectomy, and analysis of the resected specimens revealed squamous cell cancer. These findings strongly indicated metastasis from the esophageal squamous cell carcinoma, contradicting the initial diagnosis of primary breast origin. Shiraishi et al. highlighted the crucial role of histopathologic analysis and the patient's cancer history in distinguishing between primary and metastatic cancers [19]. Specifically, they noted that a breast lesion lacking ductal carcinoma or lobular carcinoma in situ might indicate a metastatic origin for the malignant breast neoplasm. 

One theory proposing to explain the metastasis of esophageal cancer to the breast suggests that it begins with the spread from the primary esophageal lesion through the draining lymphatic vessels to the thoracic duct [3]. Subsequently, there could be retrograde transmission to the breast via the internal mammary lymph nodes [3,20]. Tumor cells may access the internal mammary lymph nodes by infiltrating the intercostal vessels, which form collateral branches with the thoracic duct [3,20]. 

## Conclusions

Breast metastases originating from a primary esophageal lesion are infrequently reported. It is crucial to acknowledge that metastases from non-breast tumors can mimic primary breast cancer. Here, we present a case of primary esophageal adenocarcinoma that metastasized to the breast. Despite the rarity of this metastatic pathway, it warrants consideration in patients with breast carcinomas of unknown origin, especially in the context of a primary esophageal malignant lesion, due to its implications for treatment. Patients with a history of primary esophageal carcinoma should be considered for routine surveillance screening, including mammography, to detect potential breast metastases, with additional imaging as necessary. Our aim is to highlight this rare metastatic route and raise awareness of its possibility. 
